# The impact of a bicuspid pulmonary valve in the aortic position after arterial switch for transposition of the great arteries on neoaortic root dimension and function: a propensity score matched analysis

**DOI:** 10.1093/icvts/ivac073

**Published:** 2022-03-31

**Authors:** Bobae Jeon, Eun Seok Choi, Bo Sang Kwon, Tae-Jin Yun, Seul Gi Cha, Jae Suk Baek, Jeong Jin Yu, Chun Soo Park

**Affiliations:** 1 Division of Cardiothoracic Surgery, GangNeung Asan Hospital, Gangwon-do, Korea; 2 Division of Pediatric Cardiac Surgery, Asan Medical Center, University of Ulsan College of Medicine, Seoul, Korea; 3 Division of Pediatric Cardiology, Asan Medical Center, University of Ulsan College of Medicine, Seoul, Korea

**Keywords:** Transposition of the great arteries, Arterial switch operation, Bicuspid pulmonary valve, Neoaortic root, Neoaortic regurgitation

## Abstract

**OBJECTIVES:**

This study investigated the effect of a bicuspid pulmonary valve on neoaortic root morphology, function, and the clinical outcomes of early survivors after the arterial switch operation using propensity score matching.

**METHODS:**

From 1997 to 2018, a total of 442 patients underwent the arterial switch operation for transposition of the great arteries. After exclusion of patients who underwent a staged repair, were repaired beyond 1 year of age, died before discharge and who lacked echocardiographic data at discharge, propensity score matching was used for analysis.

**RESULTS:**

Among 352 eligible patients, 18 patients (5.1%) had a bicuspid pulmonary valve. After propensity score matching (1:4), 15 patients with bicuspid valve (bicuspid group) and 60 patients with tricuspid valve (tricuspid group) were enrolled. The median follow-up duration was 9.9 years (4 months∼22.3 years). All-cause reoperation-free survival at 10 years was 93.3% in the bicuspid group and 87.0% in the tricuspid group (*P *=* *0.839), and reoperation for neoaortic valve or root was required in 2 patients in the bicuspid group and 1 in the tricuspid group without intergroup difference. The *z*-score of the neoaortic annulus did not change in either group, although there was an increasing tendency in the z-score of the neoaortic sinus without intergroup difference (*P *=* *0.690). Deterioration in neoaortic valve function was more prominent in the bicuspid group (p = 0.028).

**CONCLUSIONS:**

The neoaortic sinus might outgrow the norm regardless of the number of neoaortic valve cusps, whereas the neoaortic annulus remained unchanged. Deterioration of valve function was more prominent in the bicuspid group, which suggests that a bicuspid valve might play a significant role in deterioration of neoaortic valve function, without an additional effect on root pathology.

**Figure:**
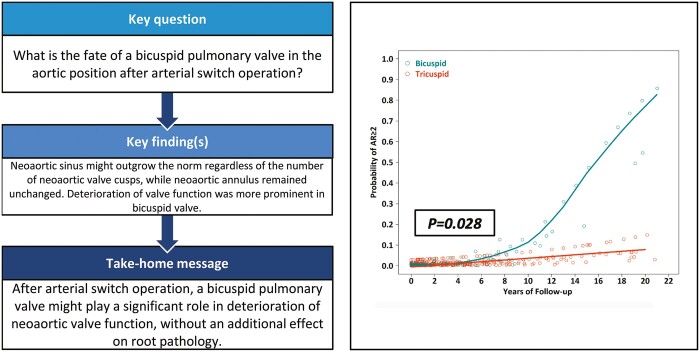

## INTRODUCTION

The arterial switch operation (ASO) has been used as a standard surgical treatment for transposition of the great arteries (TGA) since its first documented success (1). For now, the ASO can be performed with very low operative mortality in experienced centres (2, 3), although concerns still remain regarding residual problems including neoaortic root dilatation, neoaortic valve regurgitation (neoAR), coronary insufficiency and pulmonary artery stenosis in the long term.

In the normal position, a bicuspid aortic valve is known to be associated with aortic root dilatation with varying prevalence and degree of dilatation depending upon the phenotype. Moreover, a bicuspid pulmonary valve is more likely to be degenerated than a pulmonary valve with three cusps (4). Therefore, is it reasonable to ask what happens to the bicuspid pulmonary valve that becomes an aortic valve during ASO in the long term. Some studies have investigated clinical outcomes after ASO for TGA with a bicuspid pulmonary valve, changes in aortic root dimensions and progression of neoAR (4, 5). However, a small number of cases, lack of comparison among patients with different cusp morphologies and the use of a simple paired comparison for evaluating changes in neoaortic root dimensions with different time intervals are limitations of previous studies.

This study investigated the effect of a bicuspid neoaortic valve on neoaortic root morphology and function, and the long-term clinical outcomes of early survivors after ASO for TGA using propensity score matching.

## METHODS

### Ethics statement

The study was approved by Asan Medical Center institutional review board (the date and number of the institutional review board approval: 2020–09-18 and S2020-2228–0001). The requirement for informed consent was waived because of the retrospective nature of the study design.

### Patient selection and data collection

From January 1997 through December 2018, among all patients who underwent ASO for TGA, those who underwent staged repair or were repaired at an age older than 1 year were excluded because they enhanced the homogeneity of the patient cohort. For a comparison of serial changes in the morphology and function of the neoaortic root, patients who died before discharge and patients who lacked echocardiographic data at discharge that would provide baseline morphological data were also excluded. Propensity score matched data were used to compare the outcomes of early survivors according to the number of pulmonary cusps. Variables used to generate the propensity score included sex, age at operation, ventricular septal defect (VSD), Taussig-Bing anomaly and arch obstruction.

We reviewed the electronic medical records of the study cohort to collect and collate data regarding patient characteristics, anatomical details, operative details, perioperative data and follow-up data. All serial echocardiographic images for individual patients were reviewed to obtain the information about the neoaortic root dimensions, neoaortic valve function and other morphological or functional measurements. Sections that are difficult to identify accurately with echocardiography in neonatal patients, such as commissural orientation and the phenotype of the bicuspid valve, may be obtained with computed tomographic scans or magnetic resonance imaging. Preoperative computed tomography has been used at our institution for all patients undergoing surgery for complex congenital heart disease since 2015.

The data underlying this article cannot be shared publicly to protect the privacy of the individuals who participated in this study.

### Definition

Serial measurements of the neoaortic annulus and sinus were taken in the parasternal long- axis view and adjusted for body surface to calculate *z*-scores by referring to the data from the Cincinnati Children’s Hospital (6). The severity of neoAR was assessed on the basis of Doppler echocardiographic findings following the American Society of Echocardiography guidelines (7); none to trivial, 0; mild, 1; mild to moderate, 2; moderate to severe, 3; and severe, 4. Significant neoAR was defined as the degree of regurgitation equal to or greater than grade 2.

The outcomes of interest were the composite of all-cause death, major complications within 30 days after surgery and the development of significant neoAR and neoaortic root dilatation. Major complications included unplanned reoperation, cardiac arrest, arrhythmia requiring a permanent pacemaker implant, circulatory instability requiring mechanical support, acute renal failure requiring haemodialysis or haemofiltration, neurological deficit persisting at discharge and deep wound infection or mediastinitis.

### Surgical techniques

During the study period, ASO for TGA was performed by 4 surgeons. With standard aortic and bicaval venous cannulation, moderate hypothermic cardiopulmonary bypass was used for repair. Repeated doses of antegrade cold crystalloid or blood cardioplegia were administrated indirectly and directly. After transection of the main pulmonary artery, the morphological characteristics of the pulmonary valve were evaluated. None of the pulmonary valves, which were designated as future aortic valves, were ever touched in this study cohort. Decisions regarding coronary reimplantation using trapdoor incisions and before or after neoaortic reconstruction were made at the discretion of the individual surgeons. Deficient neopulmonary arteries were augmented with autologous pericardial patch with or without treatment. The Lecompte manoeuvre was used for all patients. All intracardiac procedures were accomplished before the arterial switch operation.

### Statistical analysis

The normality of variables was evaluated with the Shapiro-Wilk test. Categorical variables were presented as frequencies and percentages, and continuous variables were presented as means with standard deviations or medians with interquartile ranges (IQR) according to the distribution of the data. Before matching, categorical variables were compared with the χ^2^ or Fisher’s exact test as appropriate, and continuous variables (age) were compared using the Wilcoxon rank sum test because of its right-skewed distribution. In order to minimize the effect of selection bias in this observational study, 1:4 matching based on the propensity scores estimated with pulmonary valve cusp morphology as a dependent variable by logistic regression analysis was used. Age at operation, sex, VSD, Taussig-Bing anomaly and arch obstruction were included as confounders. The absolute standardized mean difference was used to evaluate the balance after weighting. A standardized mean difference value ≥0.1 was considered an indicator of meaningful imbalance. Conditional logistic regression analysis was used to compare the matched data. The effect of the pulmonary valve cusp morphology (bicuspid vs tricuspid) on outcomes of interest, such as any death or transplant, all-cause reoperation and reoperation related to neoaortic root or neoaortic valve was estimated using the Cox proportional hazard model. We used a mixed linear regression model with a random intercept model to evaluate the difference in serial changes of neoaortic root dimensions between groups. The effect of neoaortic valve cusp morphology on progression of neoAR was assumed by obtaining the common odds ratio (OR) derived from an ordinal logistic regression model where the expected probability of each individual was expressed as a probability curve. Statistical analyses were performed using SPSS Statistics version 22 (IBM Corp., Armonk, NY, USA), R software version 3.4.4 (R Foundation for Statistical Computing, Vienna, Austria) and the GraphPad statistical software package version 5 (GraphPad software, San Diego, CA, USA). A *P*-value less than 0.05 was considered statistically significant.

## RESULTS

### Patients characteristics

**Figure 1:**
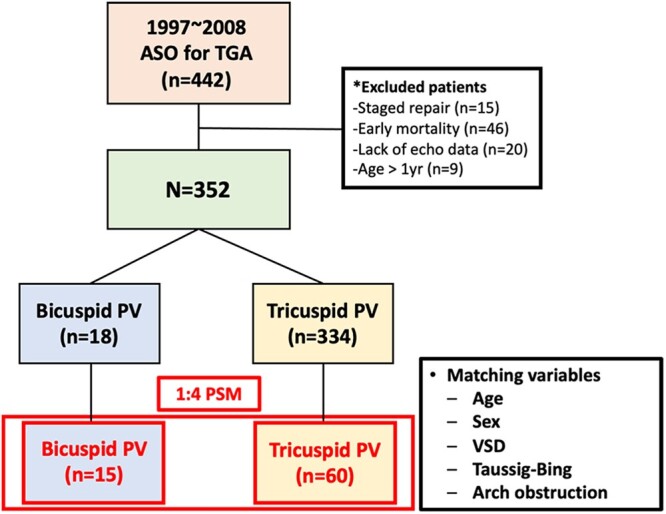
Diagram for patient selection. From January 1997 through December 2018, of 442 patients who underwent ASO for TGA, 352 patients were eligible for analysis after exclusion of the patients who underwent staged repair or were repaired at an age older than 1 year, died before discharge and lacked echocardiographic data at discharge. A total of 90 patients (15 patients in the bicuspid PV group and 60 patients in the tricuspid PV group) were included after 1:4 propensity score matching. Variables used to generate the propensity score included sex, age at operation, VSD, Taussig-Bing anomaly and arch obstruction. ASO: arterial switch operation; PSM: propensity score matching; PV: pulmonary valve; TGA: transposition of the great arteries; VSD: ventricular septal defect.

During the study period, 442 patients underwent ASO for TGA. A total of 90 patients including 15 patients who underwent staged repair, 9 patients who underwent repair who were older than 1 year of age, 46 patients who died before discharge and 20 patients for whom no echocardiographic data were available at discharge were excluded from this study. Among a total of 352 eligible patients, 18 patients (5.1%) had a bicuspid pulmonary valve. After 1:4 matching with propensity scores, 15 patients with bicuspid pulmonary valves (bicuspid group) and 60 corresponding patients with tricuspid pulmonary valves (tricuspid group) were enrolled for analysis (Fig. 1).

Table 1 shows the baseline and morphological characteristics that were used as variables for calculating propensity scores. There was no residual imbalance after propensity score matching. Table 2 shows the other baseline and morphological characteristics. There were 36 females and 39 males. Median age and body weight at initial surgical treatment, incidence of low birth weight and prematurity were comparable between the 2 groups. Thirty-six patients (36/75, 48%) were diagnosed prenatally. The relationship of the great vessels was anterior-posterior in 42 patients (56.0%), oblique in 28 patients (37.3%) and side-by-side orientation in 5 (6.7%) without significant intergroup differences (*P* = 0.096). Coronary artery anatomy was usual in 47 patients (47/75, 62.7%) without a significant intergroup difference (*P* = 0.695). Forty-five patients (45/75, 60.0%) had a VSD, and 13 patients (13/75, 17.3%) were diagnosed as having Taussig-Bing anomaly. All patients with an arch obstruction were excluded after the matching process.

**Table 1:** Covariates used for calculating propensity scores

	Before PSM			After PSM			
	Tricuspid	Bicuspid	ASD^a^	Tricuspid	Bicuspid	ASD^a^	*P-*valu*e*
	(n = 334)	(n = 18)		(n = 60)	(n = 15)		
Sex (male)	248 (74.3)	11 (61.1)	0.284	31 (51.7)	8 (53.3)	0.033	1.000
Age (days)	8 (6-13)	15 (8-23)	0.753	12 (8-17)	12 (7-20)	0.061	0.827
VSD	126 (37.7)	12 (66.7)	0.605	36 (60.0)	9 (60.0)	0.000	1.000
T-B anomaly	29 (8.7)	3 (16.7)	0.242	10 (16.7)	3 (20.0)	0.086	0.716
Arch obstruction	37 (11.1)	0 (0.0)	0.499	0 (0.0)	0 (0.0)	0.000	–

a An ASD of >0.1 is considered a meaningful imbalance.

ASD: absolute standardized difference; PSM: propensity score matching; T-B: Taussig-Bing; VSD: ventricular septal defect.

**Table 2:** Baseline and morphological characteristics

Number (%) or median (IQR)		Tricuspid (n = 60)	Bicuspid (n = 15)	*P-*value
Body weight (kg)		3.2 (2.9-3.6)	3.2 (2.8-3.5)	0.786
Low birth weight (2.5 kg)		7 (11.7)	1 (6.7)	1.000
Prematurity (37 weeks)		7 (11.7)	0 (0.0)	0.333
Era of surgery	1997∼2003	22 (36.7)	3 (20.0)	0.096
	2004∼2010	20 (33.3)	3 (20.0)	
	2011∼2018	18 (30.0)	9 (60.0)	
Coronary patterns	Usual	37 (61.7)	10 (66.7)	0.695
	Single	9 (15.0)	1 (6.7)	
	Others	14 (23.3)	4 (26.7)	
GA relationship	A-P	30 (50.0)	12 (80.0)	0.096
	Oblique	25 (41.7)	3 (20.0)	
	Side by side	5 (8.3)	0 (0.0)	

A-P: anterior-posterior; GA: great artery; IQR, interquartile range.

### Operative outcomes

The closed technique, which represents coronary reimplantation after neoaortic reconstruction, was used more frequently in the bicuspid group [9/15 (60%) in the bicuspid group vs 20/60 (33.3%) in the tricuspid group], but the difference did not reach statistical significance (*P* = 0.077) (Table 3). For a coronary reimplantation, a trapdoor incision was used in most patients [15/15 (100%) in the bicuspid group vs 58/60 (96.7%) in the tricuspid group, *P* = 1.000] (Table 3). Cardiopulmonary bypass time [197 min (IQR, 167–230 min) in the bicuspid group vs 161 min (IQR, 137–196 min) in the tricuspid group, *P* = 0.063] and aortic cross-clamp time [121 min (IQR, 91–157 min) in the bicuspid group vs 95 min (IQR, 81–115) in the tricuspid group, *P* = 0.009] were significantly longer in the bicuspid group (Table 3).

**Table 3:** Operative outcomes

Number (%) or median (IQR)		Tricuspid (n = 60)	Bicuspid (n = 15)	*P*
Order of coronary transfer	Closed	20 (33.3)	9 (60.0)	0.077
	Open	40 (66.7)	6 (40.0)	
Method of coronary transfer	Trapdoor	58 (96.7)	15 (100.0)	1.000
	Buttonhole	2 (3.3)	0 (0.0)	
CPB time (min)		161 (137-196)	197 (167-230)	0.063
ACC time(min)		95 (81-115)	121 (91-157)	0.009
Hospital stay (days)		10 (9-15)	13 (9-19)	0.240

ACC: aortic cross-clamping; CPB: cardiopulmonary bypass; IQR: interquartile range.

Major complications included residual VSD in 3 patients, mediastinal bleeding in 2 patients, residual pulmonary artery stenosis in 1 patient and pericardial effusion requiring pericardiostomy in 1 patient. The median hospital stay was not significantly different between the groups [13 days (IQR, 9–19 days) in the bicuspid group vs 10 days (IQR, 9–15 days) in the tricuspid group, *P* = 0.240] (Table 3). NeoAR was absent on the Doppler echocardiographic examination at discharge in most patients [86.7% (13/15) in bicuspid group vs 90% (54/60) in tricuspid group].

### Long-term clinical outcomes of matched early survivors

There was 1 late death (1.3%) in the tricuspid group. A full-term male baby with a birth weight of 3.5 kg who underwent ASO and VSD closure at 2 weeks after birth, died of respiratory arrest at 10.9 months postoperatively. Follow-up was completed for all patients, and the median follow-up duration was 9.9 years (range, 4 months–22.3 years). There was no significant difference in overall transplant-free survival between the groups (100% at 10 years in the bicuspid group vs 98.3% at 10 years in the tricuspid group, *P* = 0.999).

An all-cause reoperation was required in 11 patients (11/75, 14.7%) (Table 4). Freedom from all-cause reoperation at 10 years was 93.3 ± 6.4% in the bicuspid group and 87.0 ± 4.7% in the tricuspid group without a significant intergroup difference (*P* = 0.839). Reoperation for aortic valve or aortic root was required in only 3 patients during the follow-up period (3/75, 4.0%). One patient with a tricuspid pulmonary valve underwent aortic root reduction plasty at 11.9 years after repair. Another 2 patients with bicuspid pulmonary valves needed aortic valve repair for aortic valve stenosis at 10 months and aortic root reduction plasty at 19.4 years after ASO, respectively (Table 4).

**Table 4:** Reintervention or reoperation after the arterial switch operation

	Group	Age (Day)	Cause	Operation	Interval (Year)	Status
1	BPV	23	LAD compression, aortic root aneurysm	LAD ostial relocation, Aortic root reduction plasty	19.4	Alive
2	BPV	21	Valvar AS	1. Balloon AVP	0.6	Alive
				2. AVP	0.8	
3	TPV	19	VSD, subaortic ridge	VSD direct closure, subaortic membrane excision	1.0	Alive
4	TPV	19	LPA stenosis	Ballooning	1.3	Alive
5	TPV	14	Left main bronchus compression	Aortopexy	0.1	Dead
6	TPV	12	Chylothorax	Thoracic duct mass ligation	0.2	Alive
7	TPV	5	RVOTO	PV commissurotomy, pulmonary sinus augmentation	15.5	Alive
8	TPV	6	LVOTO	VSD extension, subaortic fibromuscular membrane excision	1.6	Alive
9	TPV	17	1. Supravalvar PS	1. Balloon PVP	0.7	Alive
			2. Aortic root aneurysm, RVOTO	2. Aortic root reduction plasty, RVOTR	11.9	
10	TPV	17	RVOTO	MPA widening	6.4	Alive
11	TPV	16	LVOTO	Intraventricular rebaffling, subaortic membrane resection	0.4	Alive

AS: aortic stenosis; AVP: aortic valve plasty; BPV: bicuspid pulmonary valve; LAD: left anterior descending branch; LPA: left pulmonary artery; LVOTR: left ventricular outflow tract reconstruction; MPA: main pulmonary artery; PS: pulmonary stenosis; PVP: pulmonary valve plasty; RVOTO: right ventricular outflow tract obstruction; RVOTR: right ventricular outflow tract reconstruction; TPV: tricuspid pulmonary valve; VSD: ventricular septal defect.

### Neoaortic root morphology and function

**Figure 2:**
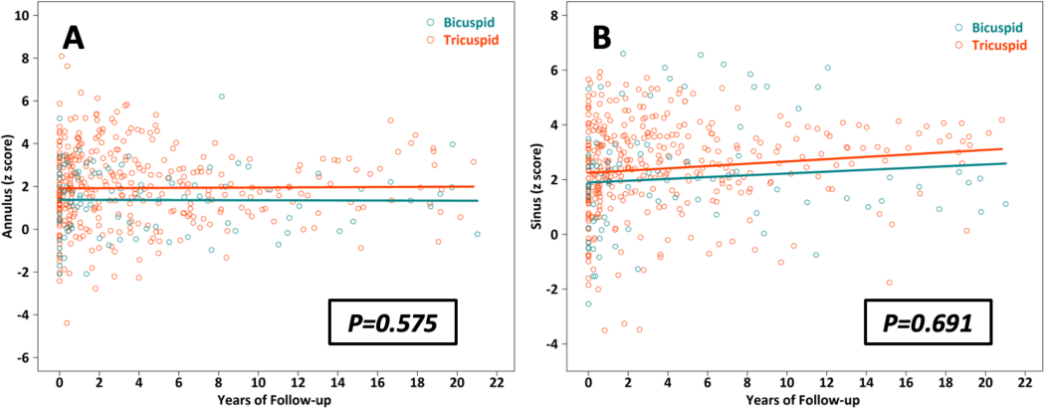
Serial changes in the dimensions of the neoaortic annulus and neoaortic sinus. (**A**) The indexed neoaortic annulus dimension (*z*-score) remained unchanged in both groups (β = 0.018, *P* = 0.563 in the bicuspid group vs β = 0.002, *P* = 0.911 in the tricuspid group) without a significant intergroup difference (*P* = 0.575). (**B**) The indexed neoaortic sinus dimension (*z*-score) increased in both groups but the change was significant only in the tricuspid group (β = 0.026, *P* = 0.285 in the bicuspid group vs β = 0.037, *P* = 0.005 in the tricuspid group). There was no significant intergroup difference (*P* = 0.691).

Baseline neoaortic root dimensions measured before discharge after ASO were similar between the groups. The indexed neoaortic annulus diameter (*z*-score) remained unchanged in both groups (β = 0.018, *P* = 0.563 in the bicuspid group vs β = 0.002, *P* = 0.911 in the tricuspid group) without a significant intergroup difference (*P* = 0.575) (Fig. 2A). The indexed neoaortic sinus diameter (*z*-score) increased in both groups, but the change was statistically significant only in the tricuspid group (β = 0.026, *P* = 0.285 in the bicuspid group vs β = 0.037, *P* = 0.005 in the tricuspid group). There was no significant intergroup difference (*P* = 0.691) (Fig. 2B)

**Figure 3:**
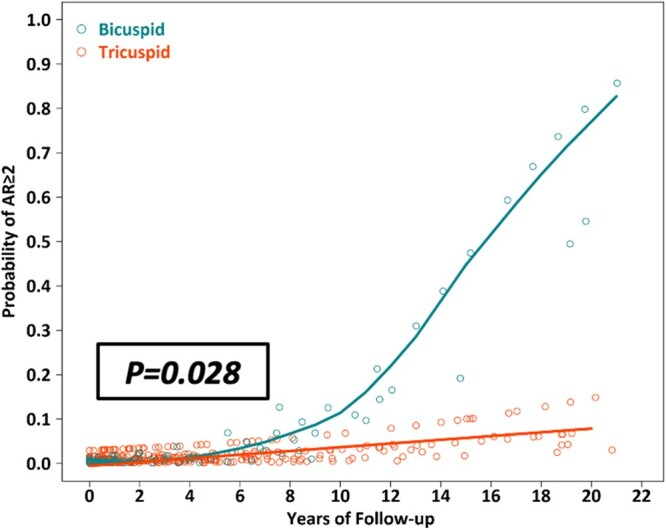
Probability of neoaortic regurgitation grade ≥ 2 during follow-up. The grade of neoaortic regurgitation worsened in both groups (common odds ratio 1.40, 95% confidence interval 1.17–1.69, *P* < 0.001 in the bicuspid group vs common odds ratio 1.12, 95% confidence interval 1.01–1.23, *P* = 0.029). The worsening of neoaortic regurgitation was more prominent in the bicuspid group (*P* = 0.028). AR: aortic regurgitation.

During follow-up, the neoAR worsened in both groups [common OR 1.40, 95% confidence interval (CI) 1.17–1.69, *P* < 0.001 in the bicuspid group vs common OR 1.12, 95% CI 1.01–1.23, *P* = 0.029]. The worsening of neoAR was more prominent in the bicuspid group (*P* = 0.028) (Fig. 3).

Neoaortic stenosis was developed in 1 patient in the bicuspid group, and reintervention was required at 7 months and 10 months after ASO (Table 4).

## DISCUSSION

The ASO is among the greatest success stories in the field of paediatric cardiac surgery. Since its first documented success in 1975 (1), it has become a standard surgical treatment for TGA. Knowledge and improved techniques were amassed by leading groups in the late 1980s (8, 9). Recent reports covering ASO for TGA have consistently noted low operative mortality (2, 3, 10), although reports about long-term concerns are still lacking. NeoAR, which might be associated with neoaortic root dilatation, is among the major concerns following ASO (11–16). Schwartz and colleagues from Boston demonstrated that post-ASO patients were at risk of neoaortic root dilatation and neoAR in the long term (11). The authors identified a previous pulmonary artery band as a risk factor for the development of neoaortic root dilatation and a previous pulmonary artery band, older age at repair and the presence of a VSD as risk factors for development of significant neoAR. It is interesting that neoaortic root dilatation seemed not to be progressive and that, in their report, surgery for neoAR was rarely required (11). In contrast, van der Palen *et al.* noted that neoaortic root dilatation was progressive and associated with the progression of neoAR (14). McMahon and colleagues (13) indicated that neoaortic root dilatation was a risk factor for the development of significant neoAR. They also identified the factors associated with neoaortic root enlargement, including previous pulmonary artery banding, the presence of VSD and Taussig-Bing anatomy.

In our study, neoaortic sinus dimensions seemed to increase over time, although only the tricuspid group showed statistical significance in this regard. Given that the bicuspid aortic valves are frequently associated with aortic dilatation partially attributable to the effect secondary to flow dynamics (17), our findings tell a different story in switched neoaortic bicuspid valves compared to the bicuspid aortic valves in the normal position; a bicuspid neoaortic valve might not have an additional effect on the neoaortic root through flow dynamics. The presence of a bicuspid pulmonary valve might render coronary artery reimplantation and aortic root reconstruction difficult; consequently, the neoaortic root could become more damaged; however, we did not observe any negative effects on aortic root morphology or function associated with technical difficulties in coronary implantation or neoaortic root manipulation. Our study also demonstrated that the growth of the neoaortic valve annulus was proportional to the somatic growth regardless of pulmonary valve morphology. Because the Leiden group noted that the growth of the neoaortic valve annulus was stabilized from 2 years to 18 years of age but started to increase again at 15 years of age (14), longer follow-up might be mandatory to draw solid conclusions.

The presence of a bicuspid pulmonary valve in TGA has not been considered an absolute contraindication for ASO unless an unrelievable left ventricular outflow tract obstruction exists (18–21). In our series, the prevalence of the bicuspid pulmonary valve among repaired TGAs was 5.1%, which is compatible with previous reports, with a prevalence ranging from 4% to 7% (4, 5, 14). Previous studies have reported that the presence of a bicuspid pulmonary valve might not be associated with additional risk for the development of significant neoAR after ASO for TGA (4, 5). However, most such studies analysed the data from small samples using simple methods, precluding a fair, comparative analysis with patients having normal tricuspid pulmonary valves. In contrast with the previous reports, we directly compared the outcomes relevant to repaired TGA among groups divided by the pulmonary valve cusp morphology. Additionally, in our study, propensity score matching could further strengthen the power of comparison between groups with different pulmonary valve morphologies.

Even though surgery for neoAR in patients who have undergone ASO for TGA has been consistently uncommon in previous studies (11, 13), likewise in our study, the occurrence of neoAR might increase if the follow-up period were extended. As previously mentioned, the neoaortic sinus growth seemed more prominent in the tricuspid group, although an increasing trend was also observed in the bicuspid group. Unlike the findings from previous studies (14, 16), in our study, the progression of neoAR was more prominent in the bicuspid group, in which the outgrowth of the neoaortic sinus was less prominent. This finding suggests that the neoaortic valve function might be affected by the neoaortic valve itself rather than by the neoaortic root pathology. Technically speaking, aortic manipulation, such as a trapdoor incision or aortic reconstruction before a coronary implant, was known to be a possible factor associated with neoAR attributed to more prominent distortion of the sinotubular junction geometry. In our study, the trapdoor incision was used in most patients (74/75, 98.7%), and aortic reconstruction was performed before a coronary implant with similar incidence in both groups; therefore, surgical factors might have little or no impact on the development of neoaortic root dilatation or neoAR.

For now, reoperations related to neoaortic valve problems are required infrequently. However, given that the worsening of neoAR was more prominent in the bicuspid group, which was demonstrated in our study, longer and close follow-up is mandatory, especially for patients with bicuspid pulmonary valves who have a repaired TGA.

Even though neoaortic valve stenosis could be a mode of valve failure in the setting of a bicuspid valve, it developed in 1 patient in the bicuspid group during follow-up in this study. The rare occurrence of neoaortic valve stenosis in our study might be attributed to a higher threshold in valve mobility and annular dimension at the time of the decision on the use of the bicuspid valve as a semilunar valve in the aortic position.

This study was limited by the inherent disadvantages of retrospective research. Furthermore, the impact of unmeasured factors on outcomes could not be excluded, although propensity adjustment might account for the measured confounders. Surgical technique and perioperative or follow-up management have changed during the study period. Throughout the study period, 4 individual surgeons who had different surgical skills and strategies with regard to coronary reimplantation and neoaortic root manipulation performed the operations, which might have affected aortic root morphology and function. Due to the small sample size, a detailed analysis regarding the morphological difference s of bicuspid pulmonary valves could not be performed. Finally, because the exclusion of patients who died before discharge could affect the clinical outcome, the readers should be careful to interpret the results of this study.

## CONCLUSIONS

The neoaortic sinus might outgrow the norm regardless of the number of neoaortic valve cusps, whereas the neoaortic annulus remained unchanged. Deterioration of aortic valve function was more prominent in the bicuspid group, which suggests that a bicuspid pulmonary valve might play a significant role in deterioration of neoaortic valve function, without an additional effect on neoaortic root pathology.

## FUNDING STATEMENT

No funding was provided for this study.

## CONFLICT OF INTEREST STATEMENT

None declared.

## AUTHOR CONTRIBUTIONS STATEMENT

Conception and design of the research and writing of the manuscript: Bobae Jeon, Chun Soo Park; Analysis and interpretation of the data: Bobae Jeon, Chun Soo Park, Eun Seok Choi, Tae-Jin Yun; Statistical analysis: Bobae Jeon, Chun Soo Park; Critical revision of the manuscript: all authors. All authors approved the final version of the manuscript.

## Notes

Presented at the 34th Annual Meeting of the European Association for Cardio-Thoracic Surgery, Barcelona, Spain, 8–10 October 2020.

## Abbreviations

ASO: Arterial switch operation
IQR: Interquartile range
neoAR: Neoaortic valve regurgitation
OR: Odds ratio
TGA: Transposition of the great arteries
VSD: Ventricular septal defect
